# Association of systemic inflammatory response index and stroke: a cross-sectional study of NHANES, 2005–2018

**DOI:** 10.3389/fneur.2025.1538352

**Published:** 2025-01-31

**Authors:** Aokai Tian, Yafang Zheng, Jing Jin, Chunyuan Huang

**Affiliations:** Liaoning University of Traditional Chinese Medicine, Shenyang, Liaoning, China

**Keywords:** systemic inflammatory response index, stroke, cross-sectional studies, NHANES, inflammations

## Abstract

**Background:**

Many inflammatory markers like systemic immune-inflammatory index (SII), neutrophil-lymphocyte ratio (NLR), and platelet-lymphocyte ratio (PLR) are associated with stroke. However, studies on the relationship between stroke and systemic inflammatory response index (SIRI) are scarce. This study was aimed at evaluating the potential association of SIRI with stroke.

**Methods:**

Our cross-sectional study included adults with sufficient SIRI and stroke data from the 2005-2018 National Health and Nutrition Examination Survey (NHANES). We used multivariable logistic regression, interaction tests, smoothed curve fitting, and subgroup analysis for assessing the independent relationship between SIRI and stroke.

**Results:**

Of 36,176 participants in this study, 1,414 (3.9%) had experienced a stroke. In a fully adjusted model, the systemic inflammatory response index displayed a significant and positive correlation with stroke (odds ratio [OR] = 1.09, 95% confidence interval [CI] = 1.04–1.15, *p* = 0.0006). Meanwhile, the odds of stroke increased by 39% in the 4th quartile, relative to the 1st quartile (OR = 1.39, 95% CI = 1.17–1.65, *p* = 0.0002). Additional interaction tests and subgroup analysis revealed that age, sex, race, education, marriage, BMI (body mass index), smoking, diabetes mellitus, hypertension, and coronary heart disease (CHD) were not positively correlated (*p* interaction >0.05). Moreover, we also found a nonlinear correlation between SIRI and stroke, with an inflection point of 2.17.

**Conclusion:**

Our results indicate that SIRI is significantly and positively related to stroke; however, its role in stroke needs to be further confirmed by large-scale prospective studies.

## 1 Introduction

Stroke is an acute cerebrovascular disorder that affects the cerebral blood supply and is caused by occlusion or hemorrhage of blood vessels within the skull. They are classified into ischemic and hemorrhagic strokes. Ischemic and hemorrhagic variants account for 80 and 20% of all strokes, respectively (1). Strokes are a substantial health concern, affecting a majority of individuals worldwide (2). However, stroke ranks fifth among factors that cause mortality in the USA. Every year, over 795,000 adults experience a stroke, and around 140,000 of them die, accounting for one-twentieth of the nation’s total deaths (3). Males are more likely to have a stroke than females (4), and the incidence is higher in women at younger ages, while in men, it increases slightly with age (5). In recent years, the stroke onset age has become younger. This might be due to several modifiable risk factors like hypertension, hyperlipidemia, diabetes mellitus, and smoking (6).

Based on peripheral blood cell counts, the Systemic Inflammatory Response Index (SIRI), which combines the numbers of neutrophils, monocytes, and lymphocytes, is an inflammatory marker that assesses the body’s inflammatory state. Notably, SIRI reflects a significant proinflammatory response induced by monocytes and neutrophils, as well as a lymphocyte-generated anti-inflammatory response (7). Numerous studies have revealed that SIRI is a novel inflammatory indicator depicting the clinical regression of various cardiovascular diseases, like subarachnoid hemorrhage (aSAH) (8, 9), malignant cerebral edema (MCE) (10), and heart valve disease (HVD) (11). It is crucial for determining the prognosis of inflammation-related diseases (12). In coronary heart disease (CHD) patients, the SIRI index shows a positive relationsip to the degree of coronary artery stenosis, making it a useful early screening indicator for assessing stenosis severity (13).

Stroke is significantly associated with SIRI. A meta-analysis demonstrated at acute ischemic stroke (AIS) patients with higher SIRI levels upon admission showed a worse functional prognosis at 3 months (14). Wei et al. (15) discovered that increased SIRI level was a risk factor associated with ischemic stroke recurrence. According to Zhang et al. (16) SIRI was related to all-cause mortality among stroke cases and had a predictive power that exceeded that of traditional inflammatory indicators such as NLR, PLR, Lymphocyte-monocyte ratio (LMR), and Red cell distribution width (RDW). Additionally, Huang et al. also explored the correlation of SIRI with clinical outcomes and prognostic indicators in hospitalized stroke patients (17).

Although the association of SIRI with stroke has been evaluated in Asian studies with small sample sizes; however, large-scale, representative research from other countries/regions is lacking. Prior research has linked SIRI to stroke incidence in patients with hypertension and asthma (18, 19). However, these studies only included hypertensive and asthmatic patients, so their results might not reflect the association between SIRI and stroke incidence in the general population. This study was aimed at investigating the association between stroke and SIRI by using the National Health and Nutrition Survey (NHANES) database.

## 2 Materials and methods

### 2.1 Study participants

**Figure 1:**
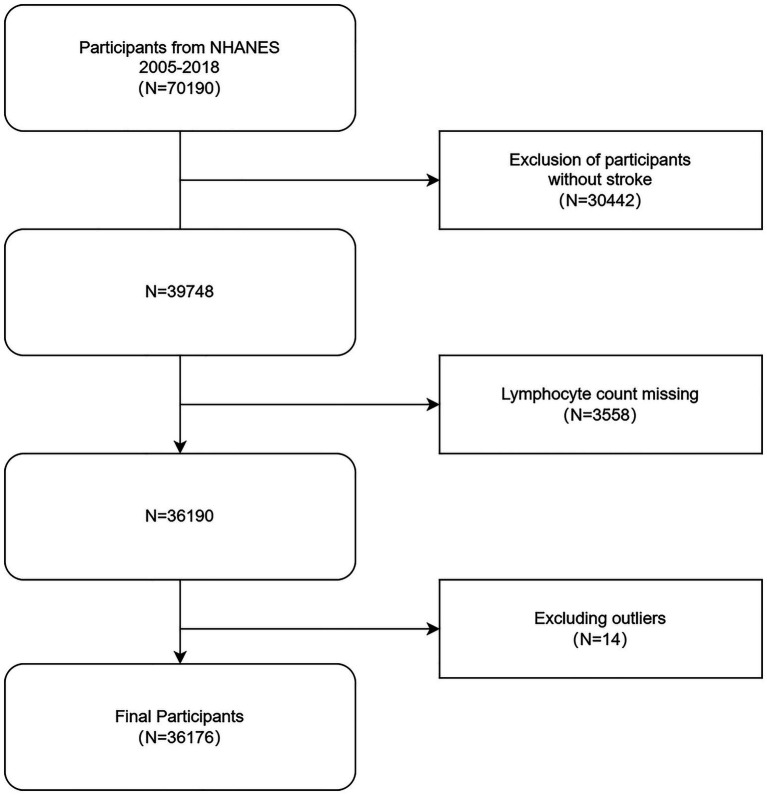
Flow chart for inclusion and exclusion of participants in the analysis. NHANES, National Health and Nutrition Examination Survey.

We used the NHANES data on stroke from 2005 to 2018. Conducted by the National Center for Health Statistics (NCHS), this continuous study covers general health and nutritional status data of US citizens. It utilizes stratified, multistage probability sampling and is conducted every 2 years for sample representativeness. We gained approval for study methods and informed consent from the NCHS Ethics Committee. NHANES data can be obtained from https://www.cdc.gov/nchs/nhanes/. Although we initially included 70,190 participants, we excluded 30,442 who lacked stroke data, 3,558 as they lacked lymphocyte counts, and 14 because of significant data outliers. All participating adults were ≥ 20 years old. Finally, we included 36,176 participants. Figure 1 displays the participants’ selection procedure.

### 2.2 Assessment of stroke

Stroke information was acquired from self-report interview data from the Medical Condition Questionnaire (MCQ). We asked the following question: “Have you ever been explicitly told by a doctor or health care professional that you have had a stroke?” Those who responded “yes” had a stroke, whereas those who responded “no” did not. The responses were used to divide participants into two categories: stroke and non-stroke groups, respectively.

### 2.3 SIRI

We designated SIRI as the exposure variable. We measured complete blood counts using the MEC autoanalyzer (Beckman Coulter MAXM; Beckman Coulter Inc.). The lymphocyte, monocyte, and neutrophil counts were represented by × 10^3^ cells/μL (19). SIRI was calculated: SIRI = (neutrophil number × monocyte number)/lymphocyte number (20).

### 2.4 Covariates

We selected variables like age, sex, race, body mass index (BMI), education, marital status, smoking, diabetes mellitus (yes/no), total cholesterol level, hypertension (yes/no), CHD (yes/no), alcohol consumption, family’s Poverty Income Ratio (PIR), taking aspirin, C-reactive protein, arthritis and chronic bronchitis as observed covariates. The four racial categories were non-Hispanic white, non-Hispanic black, Mexican American, and others. Education was divided into three types including less than high school, high school, and more than high school. BMI was simultaneously grouped into <25, 25–30, and ≥ 30 kg/m2. Marital status was categorized into living alone, married, or with a partner. Smoking was categorized into never smoking or smoking. If a person smoked 100 cigarettes in their lifetime, they were considered to be never smoking, while ≥100 cigarettes during a lifetime were considered as smoking. Self-reports from questionnaires were used to collect data regarding diabetes mellitus, CHD, hypertension, taking aspirin, arthritis and chronic bronchitis. The covariate “taking aspirin” was surveyed from NHANES 2011–2018; the covariate “C-reactive protein” was surveyed from NHANES 2005–2010.

### 2.5 Statistical analysis

Significance was set as *p* < 0.05 using R (version 4.1.3) and EmpowerStats (version 2.0). Continuous and categorical data were indicated by mean ± standard deviation (SD) and percentages, respectively. We compared differences in stroke vs. non-stroke groups by chi-square tests or t-tests for categorical and continuous data, respectively. Odds ratio (OR) with 95% confidence intervals (CI) of SIRI with stroke were generated by multivariate logistic regression. Multivariate testing generated three models: model 1 had no adjustments for any variable; model 2 had adjustments for age, sex, and ethnicity, and in model 3, all variables like were adjusted. Nonlinear correlations between SIRI and stroke were assessed using smoothed curve fitting. We evaluated subgroup analyses based on age, sex, ethnicity, education, marriage, BMI, smoking, hypertension, diabetes mellitus, CHD, taking aspirin, C-reactive protein, arthritis and chronic bronchitis for heterogeneity of associations between subgroups by adding an interaction term. Furthermore, we utilized either plurality or median interpolation for categorical and continuous data for missing values, respectively.

## 3 Results

### 3.1 Basic patient features

The average age of 36,176 participants was 49.69 ± 17.85 years. Males and females comprised 48.32 and 51.68% of the population, respectively. Participants’ mean SIRI value was 1.24 ± 0.88. The SIRI values were 1.55 ± 1.12 for stroke patients and 1.23 ± 0.87 for non-stroke patients, indicating that the higher the SIRI value, the higher the prevalence of stroke. Of the 1,414 stroke patients, 48.66% were males and 51.34% were females. Compared with subjects without a stroke, participants with stroke displayed lower total cholesterol levels, consumed less alcohol, had lower household incomes, were more non-Hispanic white, were married and cohabitating, had a higher BMI, were more hypertensive, smoked more and had lower levels of diabetes mellitus as well as coronary artery disease (CAD), More arthritis, less chronic bronchitis, taking less aspirin, higher C-reactive protein,respectively (Table 1). In addition, we compared SIRI with other biomarkers of systemic inflammation such as C-reactive protein (CRP). Due to database limitations, C-reactive protein (CRP) data were missing from 2011 to 2018, so data from SIRI and CRP from 2005 to 2010 were screened for the survey. The results showed that SIRI and CRP were significantly higher in stroke patients than in non-stroke patients.

**Table 1:** Basic characteristics of participants by stroke classification.

Characteristics	Total	Stroke YES	Stroke NO	*p*-value
	*N* = 36,176	*N* = 1,414	*N* = 34,762	
Age (years)	49.69 ± 17.85	66.43 ± 13.06	49.01 ± 17.69	<0.001
Gender, *n* (%)				0.796
Male	17,480 (48.32%)	688 (48.66%)	16,792 (48.31%)	
Female	18,696 (51.68%)	726 (51.34%)	17,970 (51.69%)	
Race, *n* (%)				<0.001
Non-Hispanic white	15,301 (42.30%)	714 (50.50%)	14,587 (41.96%)	
Non-Hispanic black	7,584 (20.96%)	383 (27.09%)	7,201 (20.72%)	
Mexican American	5,724 (15.82%)	128 (9.05%)	5,596 (16.10%)	
Other races	7,567 (20.92%)	189 (13.37%)	7,378 (21.22%)	
Education level, *n* (%)				<0.001
<High school	9,060 (25.04%)	485 (34.30%)	8,575 (24.67%)	
High school	8,266 (22.85%)	381 (26.94%)	7,885 (22.68%)	
>High school	18,850 (52.11%)	548 (38.76%)	18,302 (52.65%)	
Marital status, *n* (%)				<0.001
Married over living with a partner	21,657 (59.87%)	717 (50.71%)	20,940 (60.24%)	
live alone	14,519 (40.13%)	697 (49.29%)	13,822 (39.76%)	
BMI (kg/m^2^), *n* (%)				<0.001
<25	10,271 (28.39%)	329 (23.27%)	9,942 (28.60%)	
≥25, <30	12,284 (33.96%)	499 (35.29%)	11,785 (33.90%)	
≥30	13,621 (37.65%)	586 (41.44%)	13,035 (37.50%)	
Hypertension, *n* (%)				<0.001
No	23,205 (64.14%)	339 (23.97%)	22,866 (65.78%)	
Yes	12,971 (35.86%)	1,075 (76.03%)	11,896 (34.22%)	
Smoking, *n* (%)				<0.001
Never	20,065 (55.46%)	561 (39.67%)	19,504 (56.11%)	
Ever	16,111 (44.54%)	853 (60.33%)	15,258 (43.89%)	
Diabetes, *n* (%)				<0.001
No	30,642 (84.70%)	883 (62.45%)	29,759 (85.61%)	
Yes	5,534 (15.30%)	531 (37.55%)	5,003 (14.39%)	
Coronary heart disease, *n* (%)				<0.001
No	34,699 (95.92%)	1,161 (82.11%)	33,538 (96.48%)	
Yes	1,477 (4.08%)	253 (17.89%)	1,224 (3.52%)	
Family PIR (mean ± SD)	2.47 ± 1.55	2.06 ± 1.34	2.49 ± 1.56	<0.001
Total cholesterol (mg)	192.95 ± 41.47	183.57 ± 45.65	193.34 ± 41.25	<0.001
Alcohol consumption (mean ± SD)	2.85 ± 27.23	2.23 ± 26.57	2.87 ± 27.25	<0.001
Arthritis, *n* (%)				<0.001
Yes	9,842 (27.21%)	803 (56.79%)	9,039 (26.00%)	
No	26,334 (72.79%)	611 (43.21%)	25,723 (74.00%)	
Chronic bronchitis, *n* (%)				<0.001
Yes	2,100 (5.80%)	177 (12.52%)	1923 (5.53%)	
No	34,076 (94.20%)	1,237 (87.48%)	32,839 (94.47%)	
Taking Aspirin, *n* (%) NHANES, 2011–2018				0.779
Yes	484 (5.47%)	14 (5.88%)	456 (5.46%)	
No	8,115 (94.53%)	224 (94.12%)	7,891 (94.54%)	
SIRI (mean ± SD)	1.24 ± 0.88	1.55 ± 1.12	1.23 ± 0.87	<0.001
CRP (mg/dL) NHANES,2005–2010	0.45 ± 0.82	0.67 ± 1.31	0.44 ± 0.80	<0.001
SIRI (mean ± SD) NHANES, 2005–2010	1.23 ± 0.84	1.51 ± 1.08	1.22 ± 0.84	<0.001

PIR, Ratio of family income to poverty; BMI, body mass index; SIRI, Systemic inflammatory response index; CRP, C-reactive protein.

### 3.2 Correlation of SIRI with stroke

Table 2 shows the multivariate regression analysis of SIRI with stroke. In model 1, no covariates were adjusted. SIRI was positively correlated with stroke (OR = 1.32; 95%CI:1.26–1.38; *p* < 0.0001), suggesting a 32% increase in stroke prevalence for each 1-unit elevation of SIRI. In model 2, age, gender, and race were adjusted. SIRI was positively correlated with stroke (OR = 1.19; 95%CI:1.13–1.24; *p* < 0.0001), suggesting a 19% increase in stroke prevalence for each 1-unit elevation of SIRI. In model 3, gender; age; race; Education level; Marital status; PIR; BMI; Total cholesterol; Alcohol consumption; Hypertension; smoking; Diabetes; Coronary heart disease; arthritis and chronic bronchitis were adjusted. This positive correlation remained constant in model 3 (OR = 1.08; 95% CI: 1.03–1.14; *p* = 0.0022), suggesting a 8% increase in stroke prevalence for each 1-unit elevation of SIRI. For sensitivity analyses, we also converted SIRI from continuous data into categorical data (quartiles). The risks of suffering a stroke in model 1 were 41% higher in quartile 3, relative to quartile 1. The comparison of quartiles 1 and 2 (OR = 1.04; 95% CI: 0.87–1.24; *p* = 0.6369) did not differ statistically (*p* > 0.05). In Model 2, quartile 3 and quartile 4 had significantly increased odds of having a stroke by 32 and 74%, respectively, compared to quartile 1. The comparison of quartiles 1 and 2 (OR = 1.06; 95% CI: 0.88–1.27; *p* = 0.5401) did not differ statistically (*p* > 0.05). The risks of suffering a stroke in model 3 were 37% higher in quartile 4, relative to quartile 1. Nevertheless, the comparison of quartiles 1 and 2 (OR = 1.02; 95% CI: 0.85–1.23; *p* = 0.8301) did not differ statistically with quartile 3 (OR = 1.17; 95% CI: 0.98–1.39; *p* = 0.0908).

**Table 2:** Association of systemic inflammatory response index with stroke.

Exposure	Model 1 [OR (95% CI)]	*p*-value	Model 2 [OR (95% CI)]	*p*-value	Model 3 [OR (95% CI)]	*p*-value
SIRI (continuous)	1.32 (1.26, 1.38)	<0.0001	1.19 (1.13, 1.24)	<0.0001	1.08(1.03, 1.14)	0.0022
SIRI (quartile)						
Quartile 1	reference		reference		reference	
Quartile 2	1.04 (0.87, 1.24)	0.6369	1.06 (0.88, 1.27)	0.5401	1.02 (0.85, 1.23)	0.8301
Quartile 3	1.41 (1.20, 1.66)	<0.0001	1.32 (1.11, 1.58)	0.0015	1.17 (0.98, 1.39)	0.0908
Quartile 4	2.29 (1.96, 2.66)	<0.0001	1.74 (1.48, 2.06)	<0.0001	1.37 (1.15, 1.63)	0.0003
P for trend	1.79 (1.64, 1.96)	<0.0001	1.46 (1.33, 1.61)	<0.0001	1.25 (1.13, 1.38)	<0.0001

Model 1: no covariates were adjusted. Model 2: age, gender, and race were adjusted. Model 3: gender; age; race; Education level; Marital status; PIR; BMI; Total cholesterol.; Alcohol consumption; Hypertension; smoking; Diabetes; Coronary heart disease; arthritis and chronic bronchitis were adjusted.

PIR, Ratio of family income to poverty; BMI, body mass index; SIRI, Systemic inflammatory response index.

To further investigate the variables affecting the association between SIRI and stroke, we stratified the analysis according to age, gender, race, education, marriage, BMI, smoking, hypertension, diabetes mellitus, coronary heart disease, taking aspirin, C-reactive protein, arthritis, and chronic bronchitis (Table 3). The results showed all variables (*p* interaction >0.05), which indicated that the relationship between SIRI and stroke was not statistically different between strata. It indicates that these variables had no significant effect on the positive correlation of the relationship between SIRI and stroke (*p* interaction >0.05). However, there were significant positive associations in age, gender, other race, education level above high school, marriage, BMI ≥30 (kg/m2), having hypertension, smoking, no diabetes, no coronary heart disease, arthritis, and no chronic bronchitis.

**Table 3:** Subgroup analysis of systemic inflammatory response index and stroke.

SIRI	[OR (95%CI)]	*p* for interaction
Age		0.2246
< 60 years	1.19 (1.08, 1.31)	
≥ 60 years	1.11 (1.05, 1.17)	
Gender, *n* (%)		0.3663
Male	1.07 (1.00, 1.15)	
Female	1.12 (1.04, 1.21)	
Race, *n* (%)		0.1988
Non-Hispanic White	1.06 (0.99, 1.13)	
Non-Hispanic Black	1.09 (0.97, 1.22)	
Mexican American	1.16 (0.99, 1.35)	
Other race	1.24 (1.09, 1.43)	
Education level, *n* (%)		0.4930
<High school	1.09 (0.99, 1.19)	
High school	1.04 (0.94, 1.15)	
>High school	1.12 (1.04, 1.21)	
Marital status, *n* (%)		0.6018
Married over living with a partner	1.11 (1.03, 1.19)	
live alone	1.08 (1.01, 1.16)	
BMI (kg/m^2^)		0.5790
<25	1.07 (0.97, 1.17)	
≥25, <30	1.08 (0.99, 1.17)	
≥30	1.13 (1.04, 1.23)	
Hypertension, *n* (%)		0.5082
No	1.06 (0.95, 1.19)	
Yes	1.11 (1.04, 1.17)	
Smoking, *n* (%)		0.3617
Never	1.13 (1.04, 1.24)	
Ever	1.08 (1.01, 1.15)	
Diabetes, *n* (%)		0.1926
No	1.12 (1.05, 1.19)	
Yes	1.04 (0.96, 1.14)	
Coronary heart disease, *n* (%)		0.4421
No	1.11 (1.05, 1.18)	
Yes	1.06 (0.94, 1.18)	
Arthritis, *n* (%)		0.8697
Yes	1.09 (1.02, 1.16)	
No	1.10 (1.01, 1.19)	
Chronic bronchitis, *n* (%)		0.7984
Yes	1.08 (0.95, 1.21)	
No	1.09 (1.03, 1.16)	
Taking Aspirin, *n* (%) NHANES, 2011–2018		0.5439
Yes	0.88 (0.43, 1.80)	
No	1.10 (0.96, 1.25)	
CRP(mg/dL)NHANES, 2005–2010		0.4294
CRP < 0.45	1.03 (0.89, 1.18)	
CRP ≥ 0.45	1.11 (0.99, 1.24)	

Gender; Age; Race; Education level; Marital status; PIR; BMI; Total cholesterol.; Alcohol consumption; Hypertension; smoking; Diabetes; Coronary heart disease; arthritis and chronic bronchitis were adjusted.

PIR, Ratio of family income to poverty; BMI, body mass index; SIRI, Systemic inflammatory response index; CRP, C-reactive protein.

**Figure 2:**
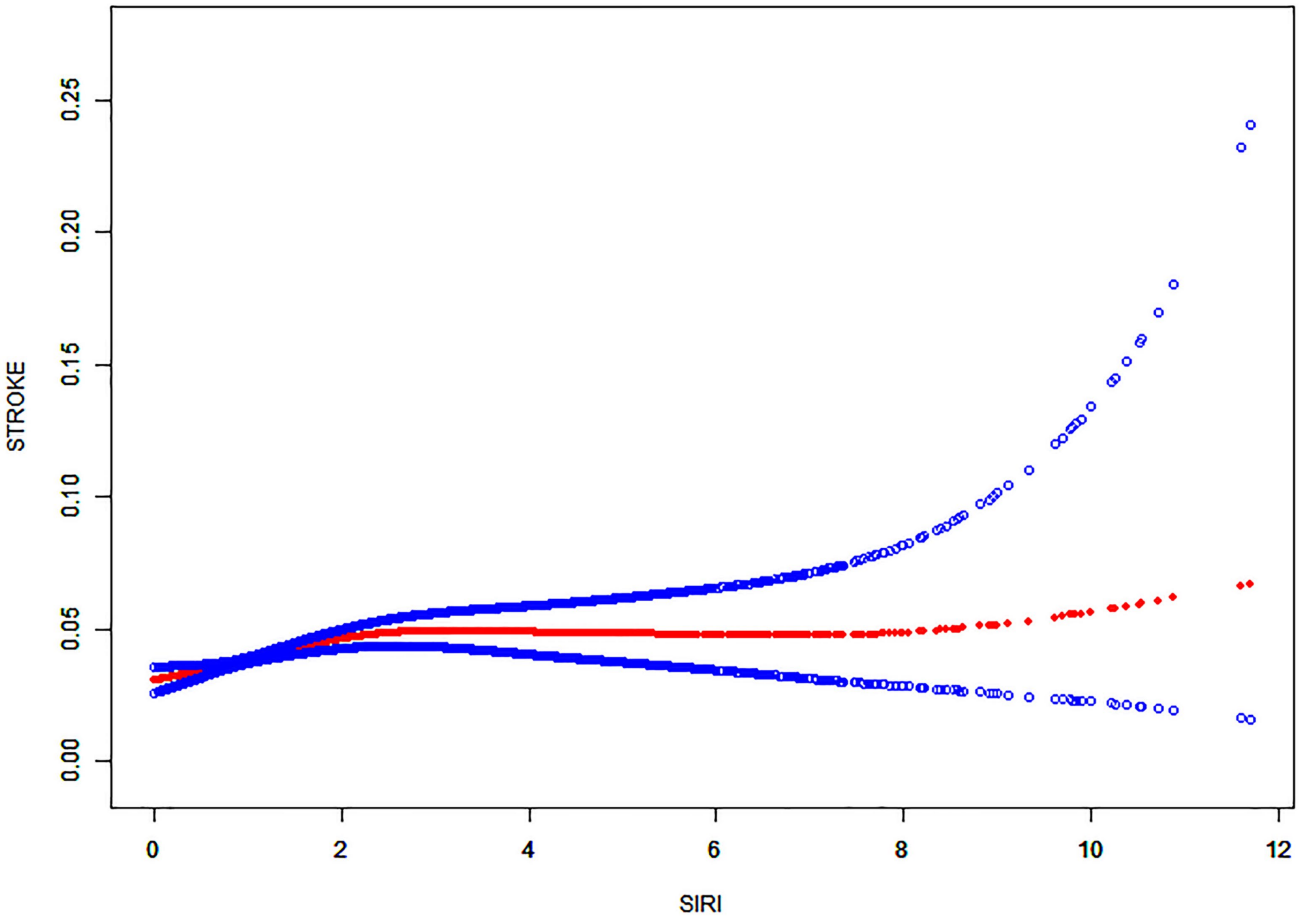
Nonlinear relationship between SIRI and stroke. The solid red line shows a smooth curve fit between the variables. The 95% confidence intervals from the fit are shown by the blue bars.

Then, using smoothed curve fitting, nonlinear associations between SIRI and stroke were shown (Figure 2). The results showed an inverted U-shaped relationship between SIRI and stroke. The association between SIRI and stroke was significantly positive before the inflection point and negative after the inflection point. Then, a threshold effect analysis of the relationship between SIRI and stroke using R (version 4.1.3) and EmpowerStats (version 2.0) yielded an inflection point of 2.17 for the nonlinear relationship between SIRI and stroke. The results showed that the risk of stroke was highest when the SIRI value was 2.17 (Table 4).The adjustment variables were age, sex, race, education, marriage, BMI, total cholesterol level, alcohol consumption, smoking, diabetes, hypertension, CHD, arthritis and chronic bronchitis.

**Table 4:** Application of Linear regression modeling to analyze the threshold effect of SIRI on stroke.

	Adjusted OR (95% CI) *p*-value
Inflection point	2.17
SIRI<2.17	1.28 (1.15, 1.43)
SIRI ≥ 2.17	0.97 (0.89, 1.06)
Log likelihood ratio	<0.001

Gender; Age; Race; Education level; Marital status; PIR; BMI; Total cholesterol.; Alcohol consumption; Hypertension; smoking; Diabetes; Coronary heart disease; arthritis and chronic bronchitis were adjusted.

SIRI, Systemic inflammatory response index.

## 4 Discussion

In this cross-sectional study including 36,176 subjects, we indicated that SIRI was positively associated with stroke. Moreover, interaction tests and subgroup analysis showed a similar relation across population settings. With an inflection point of 2.17, an inverted U-shaped correlation was observed between SIRI and stroke.

To date, SIRI is crucial for predicting cardiovascular and cerebrovascular diseases. A study by Huang (21) showed that an increased SIRI index predicted a greater stroke severity. Moreover, SIRI can be applied as a prognostic indicator for AIS patients and as an independent predictor of AIS (22–24). Conversely, higher SIRI (≥4.57) can independently predict the mortality of AIS cases (25). Additionally, SIRI is also helpful in predicting other stroke-related diseases. Lin et al. (26) found that SIRI can be conveniently used for predicting atrial fibrillation (AF) risk among ischemic stroke patients. Dan et al. (27) suggested that SIRI can effectively predict stroke-associated pneumonia (SAP), especially in AIS patients with SIRI levels (≥2.74), requiring increased vigilance for the risk of SAP. Min et al. (28) also suggested an association between elevated SIRI and post-stroke cognitive impairment (PSCI); using SIRI as a predictor could help in the early risk identification of PSCI. All these studies suggest that enhanced SIRI monitoring can improve the prognosis of stroke patients. A study which was performed by Adalet et al. (29) found that SIRI values increased in hemorrhagic stroke compared with ischemic stroke. Moreover, hemorrhagic stroke patients displayed an increased mortality rate, relative to ischemic stroke patients. This suggests that inflammation is more pronounced in hemorrhagic stroke patients. Our study confirmed a positive relationship between stroke and SIRI in model 1; this positive correlation was maintained in model 3 also. In the smoothed curve fit, the inflection point for SIRI and stroke was 2.17. According to Table 4, the association between SIRI and stroke was significantly and positively correlated on the inflection point’s left side, while a non-significant negative correlation was observed on its right side. However, the absence of correlation on the right side suggested a significant effect of SIRI and stroke threshold. In conclusion, our findings reconfirm the previous results and lay the foundation for the characterization of the impact of SIRI on stroke.

Inflammation is crucial for stroke development. It is a definitive pathway for the formation, growth, and rupture of atherosclerotic plaques (30) and initiates the development of thrombotic events (31). Early plaque formation is characterized by the migration of monocytes attached to vascular endothelium to arterial intima and transform into macrophages (32, 33). Stroke is caused by the destabilization of atherosclerotic plaques, a process closely linked to the infiltration of monocytes, macrophages, and T cells (34). Additionally, Esenwa et al. (35) showed that the association of stroke with accelerated proinflammatory disease development is associated with inflammatory response activation. Shi et al. (36) suggested that stroke impairs factors like damage-associated molecular patterns (DAMPs), responsible for localized inflammation in damaged brain regions. Xia and Peter et al. (37, 38) concluded that inflammation is essential in diverse stroke-related diseases. The possible benefits induced by anti-inflammatory targets for preventing secondary stroke, like anti-inflammatory therapy, can decrease vascular events like stroke among CAD cases. Anrather et al. (39) revealed that anti-inflammatory treatment is effective for managing ischemic stroke as they have a greater treatment window relative to reperfusion approaches. Moreover, Jalil et al. (40) discussed neuroinflammation’s effect on ischemic stroke pathology, concentrating on the blood–brain barrier (BBB) and central nervous system (CNS) interactions with peripheral immune response. They also provided an exhaustive overview of the mechanisms of inflammation-driven injury, including oxidative stress, increased MMP (matrix metalloproteinase) production, microglial cell activation, and infiltration of peripheral immune cells into the ischemic tissues. Thus, these studies corroborate the importance of inflammation in stroke development and emphasize the value of inflammatory responses for treating and preventing stroke. A novel inflammation indicator, SIRI is relatively inexpensive, easy to calculate, and exerts a superior predictive role in cardiovascular and cerebrovascular disease, compared with traditional indicators. It integrates the absolute values of peripheral blood neutrophils, monocytes, and lymphocytes, thereby reflecting the body’s immune-inflammatory status of the body more comprehensively.

The potential mechanisms behind the positive correlation between SIRI and stroke have not been well elucidated. The following are some of the possible mechanisms. A series of inflammatory responses are triggered after stroke, especially ischemic stroke (41). These responses include microglia and astrocyte activation, which in turn release cytokines, chemokines, MMPs, reactive oxygen species (ROS), as well as nitric oxide (NO). Jointly, these reactions exacerbate the brain tissue injury and disrupt BBB (42). During AIS, BBB disruption is accompanied by enhanced brain edema, in which neutrophils are initially produced in the infarct core and semidark zone regions (43). They release inflammatory factors that damage endothelial cells and basement membranes, respectively (44). Monocytes migrate early, infiltrate into the damaged area, and differentiate into macrophages in response to oxidized low-density lipoprotein (OX-LDL), capable of low density lipoprotein (LDL) phagocytosis and formation of foam cells, which in turn release various inflammatory mediators (45). Additionally, activated monocytes secrete vascular endothelial growth factor (VEGF) to stimulate vascular permeability and disrupt BBB (46). Meanwhile, lymphocyte concentration increases post-ischemic stroke and exerts a key effect on the AIS pathogenesis by modulating brain inflammation via proinflammatory cytokine production and poststroke cytotoxicity (47). Lymphocyte-endothelial cell interactions can also promote microvascular dysfunction and inflammatory factor production, resulting in neuronal cell death and BBB disruption (48). Among the T lymphocytes, helper T cells (CD4^+^ T cells) serve as biomarkers for predicting stroke outcomes. Studies have shown that CD4^+^ T cells act as immunomodulators after a stroke and display a protective effect when coping with inflammatory brain damage (49). Following a stroke, CD8^+^ T cells, another type of cytotoxic T cells, also show a marked response, growing quickly and remaining at high levels. Several cytokines and stroke-related neuroinflammation might lead to changes in CD8^+^ T-cell subsets (50). Furthermore, an increase in T-cell subsets may cause recurrence and death post-ischemic stroke (51). In acute cerebral hemorrhage (ICH), glial cell activation and cell death induce an inflammatory cascade damaging vascular and parenchymal tissues post-hemorrhage (52). The inflammatory immune response’s pathophysiological mechanisms exacerbate brain damage after ICH. Both resident immune cells (like astrocytes and microglial cells) as well as circulating immune cells, i.e., lymphocytes and neutrophils are related to inflammation (53). Furthermore, Perez et al. (54) showed that local and systemic inflammatory responses were activated with an enhanced neutrophil concentration after an ICH episode. The monocyte counts on day 7 post-admission correlated with the ICH volume. Moreover, Zhu et al. (55) showed that, in critical cerebrospinal fluid (CSF) patients, an elevated level of CD3^+^ T lymphocytes was significantly correlated with severe cerebral hemorrhage in the postoperative period. Thus, the above-mentioned studies showed changes in neutrophil, monocyte, as well as lymphocyte counts after stroke.

This study had the following strengths. Its large sample size and appropriate covariate adjustment enhanced the study’s reliability and representativeness. Nevertheless, this study had a few limitations. Our cross-sectional design did not help determine the causality. Therefore, large prospective studies are warranted to explore this relationship. Although we had adjusted many confounders, we could not rule out whether our results were influenced by other unaccounted confounders. For example, the diagnosis of stroke is defined in this paper by surveying each patient to report whether they have ever been told they had a stroke, without objective indicators such as imaging, clinical history, and other such information. This may therefore lead to bias (memory or categorization). We were unable to include these parameters in our study because they were not recorded in NHANES due to database limitations.

## 5 Conclusion

To conclude, our results indicate that SIRI is significantly correlated with stroke. As an indicator of systemic inflammatory status, SIRI is crucial for the stroke pathophysiologic process. Since we could not establish a causal relationship, future research requires additional large-scale prospective studies to confirm our findings.

## Data Availability

The datasets presented in this study can be found in online repositories. The names of the repository/repositories and accession number(s) can be found in the article/supplementary material.

## References

[1] GBD 2019 Stroke Collaborators. Global, regional, and national burden of stroke and its risk factors, 1990–2019: a systematic analysis for the global burden of disease study 2019. Lancet Neurol. (2021) 20:795–820. doi: 10.1016/S1474-4422(21)00252-0, PMID: 34487721 PMC8443449

[2] Mensah GA Wei GS Sorlie PD Fine LJ Rosenberg Y Kaufmann PG. Decline in cardiovascular mortality: possible causes and implications. Circ Res. (2017) 120:366–80. doi: 10.1161/CIRCRESAHA.116.309115, PMID: 28104770 PMC5268076

[3] Barthels D Das H. Current advances in ischemic stroke research and therapies. Biochim Biophys Acta Mol basis Dis. (2020) 1866:165260. doi: 10.1016/j.bbadis.2018.09.012, PMID: 31699365 PMC6981280

[4] Maida CD Norrito RL Daidone M Tuttolomondo A Pinto A. Neuroinflammatory mechanisms in ischemic stroke: focus on Cardioembolic stroke, background, and therapeutic approaches. Int J Mol Sci. (2020) 21:6454. doi: 10.3390/ijms21186454, PMID: 32899616 PMC7555650

[5] Kuriakose D Xiao Z. Pathophysiology and treatment of stroke: present status and future perspectives. Int J Mol Sci. (2020) 21:7609. doi: 10.3390/ijms21207609, PMID: 33076218 PMC7589849

[6] Bhatt N Malik AM Chaturvedi S. Stroke in young adults. Neurol Clin Pract. (2018) 8:501–6. doi: 10.1212/CPJ.0000000000000522, PMID: 30588380 PMC6294527

[7] Yi HJ Sung JH Lee DH. Systemic inflammation response index and systemic immune-inflammation index are associated with clinical outcomes in patients treated with mechanical Thrombectomy for large artery occlusion. World Neurosurg. (2021) 153:e282–9. doi: 10.1016/j.wneu.2021.06.113, PMID: 34217857

[8] Yun S Yi HJ Lee DH Sung JH. Systemic inflammation response index and systemic immune-inflammation index for predicting the prognosis of patients with aneurysmal subarachnoid hemorrhage. J Stroke Cerebrovasc Dis. (2021) 30:105861. doi: 10.1016/j.jstrokecerebrovasdis.2021.105861, PMID: 34034125

[9] Qin Y Liu L Zhao S Wang W Han M Dong S. Blood inflammatory biomarkers predict in-hospital pneumonia after endovascular treatment of aneurysm in patients with aneurysmal subarachoid hemorrhage. Neurosurg Rev. (2023) 46:171. doi: 10.1007/s10143-023-02082-5, PMID: 37436536

[10] Ji Y Xu X Wu K Sun Y Wang H Guo Y. Prognosis of ischemic stroke patients undergoing endovascular Thrombectomy is influenced by systemic inflammatory index through malignant brain edema. Clin Interv Aging. (2022) 17:1001–12. doi: 10.2147/CIA.S365553, PMID: 35814350 PMC9259057

[11] Karaçalılar M Demir M. A novel predictor in patients undergoing heart valve surgery: systemic inflammation response index: a single center cross-sectional study. Eur Rev Med Pharmacol Sci. (2023) 27:1016–22. doi: 10.26355/eurrev_202302_31196, PMID: 36808347

[12] Zuo R Zhu F Zhang C Ma J Chen J Yue P. The response prediction and prognostic values of systemic inflammation response index in patients with advanced lung adenocarcinoma. Thorac Cancer. (2023) 14:1500–11. doi: 10.1111/1759-7714.14893, PMID: 37128769 PMC10234781

[13] He T Luo Y Wan J Hou L Su K Zhao J. Analysis of the correlation between the systemic inflammatory response index and the severity of coronary vasculopathy. Biomol Biomed. (2024) 24:1726–34. doi: 10.17305/bb.2024.10747, PMID: 38907736 PMC11496849

[14] Han Y Lin N. Systemic inflammatory response index and the short-term functional outcome of patients with acute ischemic stroke: a Meta-analysis. Neurol Ther. (2024) 13:1431–51. doi: 10.1007/s40120-024-00645-2, PMID: 39117893 PMC11393365

[15] Wei X Cheng J Zhang L Xu R Zhang W. Association of systemic inflammatory response index and plaque characteristics with the severity and recurrence of cerebral ischemic events. J Stroke Cerebrovasc Dis. (2024) 33:107558. doi: 10.1016/j.jstrokecerebrovasdis.2024.107558, PMID: 38262100

[16] Zhang Y Xing Z Zhou K Jiang S. The predictive role of systemic inflammation response index (SIRI) in the prognosis of stroke patients. Clin Interv Aging. (2021) 16:1997–2007. doi: 10.2147/CIA.S339221, PMID: 34880606 PMC8645951

[17] Huang YW Zhang Y Feng C An YH Li ZP Yin XS. Systemic inflammation response index as a clinical outcome evaluating tool and prognostic indicator for hospitalized stroke patients: a systematic review and meta-analysis. Eur J Med Res. (2023) 28:474. doi: 10.1186/s40001-023-01446-3, PMID: 37915088 PMC10621190

[18] Chen J Luo C Tan D Li Y. J-shaped associations of pan-immune-inflammation value and systemic inflammation response index with stroke among American adults with hypertension: evidence from NHANES 1999–2020. Front Neurol. (2024) 15:1417863. doi: 10.3389/fneur.2024.1417863, PMID: 39144717 PMC11322096

[19] Cheng W Bu X Xu C Wen G Kong F Pan H. Higher systemic immune-inflammation index and systemic inflammation response index levels are associated with stroke prevalence in the asthmatic population: a cross-sectional analysis of the NHANES 1999-2018. Front Immunol. (2023) 14:1191130. doi: 10.3389/fimmu.2023.1191130, PMID: 37600830 PMC10436559

[20] Qi Q Zhuang L Shen Y Geng Y Yu S Chen H. A novel systemic inflammation response index (SIRI) for predicting the survival of patients with pancreatic cancer after chemotherapy. Cancer. (2016) 122:2158–67. doi: 10.1002/cncr.30057, PMID: 27152949

[21] Huang L. Increased systemic immune-inflammation index predicts disease severity and functional outcome in acute ischemic stroke patients. Neurologist. (2022) 28:32–8. doi: 10.1097/NRL.0000000000000464, PMID: 36125980 PMC9812414

[22] Zhou Y Zhang Y Cui M Zhang Y Shang X. Prognostic value of the systemic inflammation response index in patients with acute ischemic stroke. Brain Behav. (2022) 12:e2619. doi: 10.1002/brb3.2619, PMID: 35588444 PMC9226852

[23] Chen YF Qi S Yu ZJ Li JT Qian TT Zeng Y. Systemic inflammation response index predicts clinical outcomes in patients with acute ischemic stroke (AIS) after the treatment of intravenous thrombolysis. Neurologist. (2023) 28:355–61. doi: 10.1097/NRL.0000000000000492, PMID: 37027178 PMC10627531

[24] Han J Yang L Lou Z Zhu Y. Association between systemic immune-inflammation index and systemic inflammation response index and outcomes of acute ischemic stroke: a systematic review and Meta-analysis. Ann Indian Acad Neurol. (2023) 26:655–62. doi: 10.4103/aian.aian_85_23, PMID: 38022472 PMC10666886

[25] Dang H Mao W Wang S Sha J Lu M Cong L. Systemic inflammation response index as a prognostic predictor in patients with acute ischemic stroke: a propensity score matching analysis. Front Neurol. (2022) 13:1049241. doi: 10.3389/fneur.2022.1049241, PMID: 36703636 PMC9871574

[26] Lin KB Fan FH Cai MQ Yu Y Fu CL Ding LY. Systemic immune inflammation index and system inflammation response index are potential biomarkers of atrial fibrillation among the patients presenting with ischemic stroke. Eur J Med Res. (2022) 27:106. doi: 10.1186/s40001-022-00733-9, PMID: 35780134 PMC9250264

[27] Yan D Dai C Xu R Huang Q Ren W. Predictive ability of systemic inflammation response index for the risk of pneumonia in patients with acute ischemic stroke. Gerontology. (2023) 69:181–8. doi: 10.1159/000524759, PMID: 35584610

[28] Chu M Luo Y Wang D Liu Z Niu H Wu X. Prediction of poststroke cognitive impairment based on the systemic inflammatory response index. Brain Behav. (2024) 14:e3372. doi: 10.1002/brb3.3372, PMID: 38376025 PMC10771225

[29] Göçmen A Gesoglu DT. The aggregate index of systemic inflammation as a predictor of mortality in stroke patients. Cureus. (2024) 16:e64007. doi: 10.7759/cureus.64007 39109115 PMC11301770

[30] Chaturvedi S De Marchis GM. Inflammatory biomarkers and stroke subtype: An important new frontier. Neurology. (2024) 102:e208098. doi: 10.1212/WNL.0000000000208098, PMID: 38165352

[31] Kelly PJ Lemmens R Tsivgoulis G. Inflammation and stroke risk: a new target for prevention. Stroke. (2021) 52:2697–706. doi: 10.1161/STROKEAHA.121.034388, PMID: 34162215

[32] Soehnlein O Libby P. Targeting inflammation in atherosclerosis - from experimental insights to the clinic. Nat Rev Drug Discov. (2021) 20:589–610. doi: 10.1038/s41573-021-00198-1, PMID: 33976384 PMC8112476

[33] Bäck M Yurdagul A Tabas I Öörni K Kovanen PT. Inflammation and its resolution in atherosclerosis: mediators and therapeutic opportunities. Nat Rev Cardiol. (2019) 16:389–406. doi: 10.1038/s41569-019-0169-2, PMID: 30846875 PMC6727648

[34] Spagnoli LG Mauriello A Sangiorgi G Fratoni S Bonanno E Schwartz RS. Extracranial thrombotically active carotid plaque as a risk factor for ischemic stroke. JAMA. (2004) 292:1845–52. doi: 10.1001/jama.292.15.1845, PMID: 15494582

[35] Esenwa CC Elkind MS. Inflammatory risk factors, biomarkers and associated therapy in ischaemic stroke. Nat Rev Neurol. (2016) 12:594–604. doi: 10.1038/nrneurol.2016.125 27615422

[36] Shi K Tian DC Li ZG Ducruet AF Lawton MT Shi FD. Global brain inflammation in stroke. Lancet Neurol. (2019) 18:1058–66. doi: 10.1016/S1474-4422(19)30078-X, PMID: 31296369

[37] Xia M Yang X Qian C. Meta-analysis evaluating the utility of colchicine in secondary prevention of coronary artery disease. Am J Cardiol. (2021) 140:33–8. doi: 10.1016/j.amjcard.2020.10.043, PMID: 33137319

[38] Thompson PL Nidorf SM. Anti-inflammatory therapy with canakinumab for atherosclerotic disease: lessons from the CANTOS trial. J Thorac Dis. (2018) 10:695–8. doi: 10.21037/jtd.2018.01.119, PMID: 29607136 PMC5864670

[39] Anrather J Iadecola C. Inflammation and stroke: An overview. Neurotherapeutics. (2016) 13:661–70. doi: 10.1007/s13311-016-0483-x, PMID: 27730544 PMC5081118

[40] Candelario-Jalil E Dijkhuizen RM Magnus T. Neuroinflammation, stroke, blood-brain barrier dysfunction, and imaging modalities. Stroke. (2022) 53:1473–86. doi: 10.1161/STROKEAHA.122.036946, PMID: 35387495 PMC9038693

[41] Iadecola C Buckwalter MS Anrather J. Immune responses to stroke: mechanisms, modulation, and therapeutic potential. J Clin Invest. (2020) 130:2777–88. doi: 10.1172/JCI135530, PMID: 32391806 PMC7260029

[42] Liang Z Lou Y Hao Y Li H Feng J Liu S. The relationship of astrocytes and microglia with different stages of ischemic stroke. Curr Neuropharmacol. (2023) 21:2465–80. doi: 10.2174/1570159X21666230718104634, PMID: 37464832 PMC10616922

[43] Iadecola C Anrather J. The immunology of stroke: from mechanisms to translation. Nat Med. (2011) 17:796–808. doi: 10.1038/nm.2399, PMID: 21738161 PMC3137275

[44] Santos-Lima B Pietronigro EC Terrabuio E Zenaro E Constantin G. The role of neutrophils in the dysfunction of central nervous system barriers. Front Aging Neurosci. (2022) 14:965169. doi: 10.3389/fnagi.2022.965169, PMID: 36034148 PMC9404376

[45] Anselmo AC Gilbert JB Kumar S Gupta V Cohen RE Rubner MF. Monocyte-mediated delivery of polymeric backpacks to inflamed tissues: a generalized strategy to deliver drugs to treat inflammation. J Control Release. (2015) 199:29–36. doi: 10.1016/j.jconrel.2014.11.027, PMID: 25481443

[46] Xie L Zhang S Huang L Peng Z Lu H He Q. Single-cell RNA sequencing of peripheral blood reveals that monocytes with high cathepsin S expression aggravate cerebral ischemia-reperfusion injury. Brain Behav Immun. (2023) 107:330–44. doi: 10.1016/j.bbi.2022.11.001, PMID: 36371010

[47] Kawabori M Yenari MA. Inflammatory responses in brain ischemia. Curr Med Chem. (2015) 22:1258–77. doi: 10.2174/0929867322666150209154036, PMID: 25666795 PMC5568039

[48] Wang R Zhu Y Liu Z Chang L Bai X Kang L. Neutrophil extracellular traps promote tPA-induced brain hemorrhage via cGAS in mice with stroke. Blood. (2021) 138:91–103. doi: 10.1182/blood.2020008913, PMID: 33881503 PMC8288643

[49] Li S Huang Y Liu Y Rocha M Li X Wei P. Change and predictive ability of circulating immunoregulatory lymphocytes in long-term outcomes of acute ischemic stroke. J Cereb Blood Flow Metab. (2021) 41:2280–94. doi: 10.1177/0271678X21995694, PMID: 33641517 PMC8393304

[50] Bodhankar S Chen Y Lapato A Vandenbark AA Murphy SJ Saugstad JA. Regulatory CD8(+)CD122 (+) T-cells predominate in CNS after treatment of experimental stroke in male mice with IL-10-secreting B-cells. Metab Brain Dis. (2015) 30:911–24. doi: 10.1007/s11011-014-9639-8, PMID: 25537181 PMC4481189

[51] Nadareishvili ZG Li H Wright V Maric D Warach S Hallenbeck JM. Elevated pro-inflammatory CD4+CD28- lymphocytes and stroke recurrence and death. Neurology. (2004) 63:1446–51. doi: 10.1212/01.WNL.0000142260.61443.7C, PMID: 15505163

[52] Xu X Li Y Chen S Wu X Li J Li G. Mechanism and application of immune interventions in intracerebral haemorrhage. Expert Rev Mol Med. (2024) 26:e22. doi: 10.1017/erm.2024.22, PMID: 39375846 PMC11488334

[53] Ardic AF Ardic N. Role of neutrophils as therapeutic targets in intracerebral hemorrhage. Ther Innov Regul Sci. (2024) 58:807–16. doi: 10.1007/s43441-024-00668-9, PMID: 38753134

[54] Tapia-Pérez JH Karagianis D Zilke R Koufuglou V Bondar I Schneider T. Assessment of systemic cellular inflammatory response after spontaneous intracerebral hemorrhage. Clin Neurol Neurosurg. (2016) 150:72–9. doi: 10.1016/j.clineuro.2016.07.010 27611984

[55] Zhu C Zhang Y Li W Yan L Shan X Hao Y. High expression of CD3+T-lymphocytes in cerebrospinal fluid increases the risk of critical cerebral hemorrhage with systemic inflammatory response syndrome (SIRS) after surgery. Clin Chim Acta. (2024) 565:119997. doi: 10.1016/j.cca.2024.119997, PMID: 39401654

